# Acute Glomerulonephritis in a Child with *Chlamydia pneumoniae* Infection: A Case Report

**DOI:** 10.1155/2013/570921

**Published:** 2013-07-18

**Authors:** Giovanna Vitaliti, Raffaele Falsaperla, Leandra Giunta, Giuseppina Spataro, Venerando Rapisarda, Mario Velardita, Giuseppe Nunnari, Piero Pavone

**Affiliations:** ^1^Pediatric Acute and Emergency Operative Unit, Policlinico-Vittorio-Emanuele University Hospital, University of Catania, Via Plebiscito 628, 95100 Catania, Italy; ^2^Pediatric Consultant of the Regional Institution, ASP3, 95100 Catania, Italy; ^3^Department of Internal Medicine and Systemic Diseases, Division of Medicine for Job, University of Catania, 95100 Catania, Italy; ^4^Pediatric Operative Unit, Gravina and Santo Pietro of Caltagirone Hospital, 95100 Catania, Italy; ^5^Division of Infectious Diseases, Thomas Jefferson University, Philadelphia, PA, USA

## Abstract

*Background*. Infectious diseases seem to be an important and independent risk factor for renal failure, but the underlying mechanism of renal involvement during some kinds of infectious diseases is still unclear, even if the literature data report immunomediated and/or autoimmune mechanisms to explain the pathogenic relationship between the two diseases. In paediatric patients, *Chlamydia pneumoniae* is a rare cause of renal complications and it may manifest in several ways, mainly involving the respiratory system, even if also renal and glomerulalr complications, have been described. *Case Diagnosis/Treatment*. Herein we report a case of a 3-year-old child who developed an acute glomerulonephritis that was chronologically, clinically, and biologically related to a previous *Chlamydia pneumoniae* infection. On our knowledge, in the literature it is the youngest patient with renal involvement during course of *Chlamydia pneumoniae* infection ever reported. *Conclusions*. The present case supports the hypothesis of a rather close causal relationship between this infective agent and renal and glomerular symptoms occurred in this child, during an acute episode of respiratory disease.

## 1. Introduction

The literature data have shown that infectious diseases seem to represent an underestimated causative factor of renal and glomerular disorders both in childhood and in adulthood [1, 2]. The question is what kind of infective agent, and physiopathologic relationship may exist between infection and renal involvement.

*Chlamydia pneumoniae* (CP) is a common cause of respiratory disease both in adults and in children, and although the disease is generally mild and self-limiting, it may occasionally be associated with a variety of extrapulmonary conditions. Usually they occur not less than 3 days after onset of CP respiratory disease (parainfectious type) and up to 2-3 weeks after respiratory disease subsides (postinfectious type) with a heterogeneous spectrum [1–7].

To date the glomerulonephritis after a *C. pneumoniae* infection has been reported only in few adult and in quite fewer children, and a probable autoimmune mechanism has been proposed, even if until now the pathophysiology remains not yet completely clear.

Here we report a 3-year-old child who had an acute glomerulonephritis (AGN) after *Chlamydia pneumoniae* infection.

## 2. Case Report

A 3-year-old female child was admitted to our Pediatric Acute and Emergency Unit, Policlinico-Vittorio-Emanuele Hospital, University of Catania, Italy, for respiratory distress, dyspnea, and fever.

Six months before, the child was previously admitted to the Division of Paediatrics of the San Paolo Hospital in Milano for right bronchopneumonia. In that occasion, serological test showed the presence of *Mycoplasma pneumoniae* that was successfully treated with azitromicin.

The patient was a third-born to healthy nonconsanguineous parents. She was born at 39 weeks of gestation by normal delivery after uncomplicated pregnancy. At birth her weight was 3650 g, length 51 cm, and head circumference 35 cm. The perinatal period was uneventful, as far as her physical and neurological development. 

When she was admitted to our unit, the mother referred that the child had persistent fever from 7 days, treated with ceftibuten for 5 days, without improvement of her symptoms. Thus, for the persistence of her fever, she was hospitalized. At physical examination at admission her auxological parameters were weight 15.300 kg (50th percentile), length 97 cm (50th percentile), and head circumference 51 cm (50th percentile). Her body temperature was 37.8°C, heart rate 85/min, blood pressure 140/70 mmHg, and respiratory rate 21/min. She presented bibasilar rales, but all the rest of her physical evaluation was normal, including cardiac exam. Chest X-ray showed a left lower lobe *pneumoniae*. The cranial nerves were normal, as well as ocular funduscopy examination and cerebellar tests. She also underwent a sweat chloride test evaluation, EKG, and cardiac echocardiography that were all normal. 

Coagulation baseline study showed normal values for prothrombin time, partial thromboplastin time, fibrinogen, fibrin D-dimer level, protein S, protein C, factor V, factor VIII, and antithrombin III. Rheumatoid factor, ASLO titer, immunoglobulins, IgA and IgG antigliadin antibodies (AGA), IgA antiendomisium antibodies (EMA). Anticardiolipin antibodies, antiphospholipid antibodies, antinuclear antibodies, and anti-DNA were absent. Complete blood count showed 22.100 white cells/mm^3^ with 77% segmented neutrophils. Both C-reactive protein (CRP) (66.6 mg/dL) and erythrocyte sedimentation rate were increased. Normal values were recorded for hematocrit, platelet count, glucose, sodium, potassium, creatinine, plasma urea, creatine kinase, pancreas, and liver enzyme levels. Throat culture was negative. Serum amino acid (including homocysteine), serum ammonia, and blood lactic acid were also normal. Tests for hepatitis viruses and HIV-1 were negative. Rapid viral diagnostic tests for influenza, parainfluenza, and respiratory syncytial viruses were negative. Titers herpes simplex virus types 1 and 2, measles, cytomegalovirus, influenza virus, mumps, *Mycoplasma*, and *Borrelia burgdorferi* were all negative. Cold agglutinin values were positive. The serum titer of antibodies to *Chlamydia pneumonia*, tested by a microparticle agglutination assay was 1 : 310. Serum immunoblot assay revealed positive IgM (180) and IgG (330) against *Chlamydia pneumoniae*. 

A diagnosis of pneumonia from *Chlamydia pneumoniae* infection was made, and a therapy with antibiotics (ceftriaxone IV and oral Claritromycin) was started. Within 7 days she developed oliguria and stranguria, periorbital oedema. The day after she presented haematoproteinuria and peripheral oedema. At this time laboratory tests were as follows: white cells count 16.700; CRP 42.2 mg/dL; creatinine 1.75 mg/dL, urea 59 mg/dL; IgG 1.100 mg/dL, IgA 653 mg/dL, and IgM 89 mg/dL, complement fraction 3 (C3) 100 mg/dL complement fraction 4 (C4) 40 mg/dL; CH50 30.2 U/mL; C1 q inhibitor 4.5 mg/mL (nv less 3.0 mg/mL) serum total protein 7.1 g/dL, albumin 4.2 g/dL. Urinalysis revealed mild proteinuria (0.89 g/day) and hematuria (>100 RBC per high power field) with granular and hyaline casts. The anti-*C. pneumoniae* IgM and IgA Abs showed significantly high titers of 1.17 U and 3.06 U, respectively (nr < 0.90 U). IgG Abs were absent. No bacteria were isolated from throat and sputum cultures neither from urino-culture. Since the child did not show any clinical improvement, with persistence of oedema, the parents decided to go to another hospital where the child underwent to the percutaneous renal biopsy. The light microscopy of the renal biopsy samples revealed 14 glomeruli, one sclerosed. The remaining glomeruli showed an increased of mesangial cells and matrix and the presence of many neutrophils within the glomerular tuft. Three glomeruli had cellular crescent formations. Bowmann's capsule was disrupted in two glomeruli. There were focal and segmental glomerular necrosis and intracapillary fibrin or hyaline thrombi. Focal tubular atrophy and moderate interstitial damage associated with mononuclear cell infiltration were observed. There was no renal arteriolitis with fibrinoid necrosis. Immunofluorescence microscopy (IF) revealed granular staining for IgG, IgA, and C3 along the capillary walls and in mesangial areas. There was no C1 q inhibitor depositions in the glomeruli. Paramesangial deposits were detected by electron microscopy (EM), but no hump-shaped deposits were observed. Based on these findings the pathological diagnosis was mesangial and endocapillary proliferative glomerulonephritis. The identification by nested PCR of nucleic acids in the extract obtained from the renal biopsy sample was positive for *Chlamydia pneumoniae*, negative for Parvovirus B19 and *Mycoplasma pneumoniae*.

The patient came back to our observation after 15 days; the patient was in good health, with no more signs of oedema and an improvement of her renal function and normalization of both hypergammaglobulinemia and CRP. Two and six months later the routine analysis, among with urinanalysis, were normal.

## 3. Discussion

Acute glomerulonephritis (AGN) is associated with different kinds of infections, mainly *Staphylococcus* or Beta hemolitic streptococcus Group A infections, although, *Mycoplasma pneumoniae*, pneumococcus, varicella zoster, Coxiella Burnetii, and *Chlamydia pneumoniae* have also been reported to induce AGN [1–6].

*Chlamydia pneumoniae* infection is associated with several manifestations from the central nervous system to the renal involvement [1–8]. It may play a role in the development of atherosclerosis and be involved in coronary artery and cerebrovascular diseases [8, 9]. Probably *C. pneumoniae* seems to induce immune dysregulation and to play a key role in the pathogenesis of renal and glomerular insult [1–5]. It has been shown that *C. pneumoniae* organisms can survive and multiply within, not only macrophage, but also polymorphonuclear neutrophils (PMN) [10], thus leading to a persistent infection and probably to an autoimmunity process, enhancing autoantibodies production such as antineutrophil cytoplasm Ab (ANCA) [11]. In addition, *C. pneumoniae* seems to be responsible for vascular endothelial cells damage at the basis of atherosclerosis. *C. pneumonia-*infected macrophages adhere to the endothelium and migrate to the subendothelium in atherosclerotic lesion, releasing cytokines and growth factor synthesis, which upregulate endothelial cell adhesion molecules, leading to increased leucocyte adhesion [12]. All these components, including macrophage infiltration, cytokine release, upregulation of adhesion molecules, and leucocyte adhesion, have been demonstrated to be indispensable to the pathogenesis of myeloperoxidase antineutrophil cytoplasmic autoantibody-associated glomerulonephritis (MPO-ANCA-associated GN), explaining the renal pathogenic involvement during a *C. pneumoniae* infection [13]. Furthermore, *C. pneumoniae* produces chlamydial heat shock proteins (cHSPs) in infected cell macrophages and elicits a hypersensitivity reaction of the host, resulting in severe endothelial injury [11]. It is likely that the focal inflammatory reaction would be strongly enhanced resulting in necrotizing vasculitis in the presence of circulating ANCA, the production of which may be the result of interactions between T cells and B cells activated by microbial superantigens [11]. 

In the literature there are several reports on renal involvement after *C. pneumoniae* infection, even if there are still few data on this regard. In 1979, Byrom and Walls [8] described for the first time a case of acute renal failure (ARF) caused by *Chlamydia psittaci*. Since then, other cases of renal involvement and chlamydial infections have been reported [3, 11, 14, 15]. 

The patient we report showed a clinically, chronologically, and biological relation between respiratory infection and glomerunephritis. Just few weeks before the onset of glomerular symptoms, the girl suffered of fever, dyspnea and respiratory distress. A diagnosis of glomerulonephritis secondary to *Chlamydia pneumoniae* infection was made according to clinical and the nested PCR from the sample of the child's renal biopsy. 

Extrapulmonar symptoms in children with *Chlamydia pneumoniae* infections may begin with renal and glomerular involvement [1–3]. Although renal involvement is rare especially in children, a mechanism due to an autoimmune event triggered by a preceding bacterial or *Chlamydia* infection can be hypothesized [4–8].

Iyode et al. [2] described a case of a patient with rapidly progressive glomerulonephritis after *Chlamydia pneumoniae* infection. An 88-year-old woman who had been infected with *C. pneumoniae *infection was admitted to hospital after two months, with complaints of dyspnea and generalized edema. Laboratory tests revealed the presence of ARF, polyclonal hypergammaglobulinemia, highly increased level of CRP, and hamatoproteinuria. A renal biopsy revealed mesangial and endocapillary proliferative glomerulonephritis with crescents. These clinicopathological features were similar to those of superantigen-associated glomerulonephritis after methicillin-resistant *Staphylococcus aureus* infection. The authors suggested that the superantigenic mechanism is one of the possible pathomechanisms of this glomerulonephritis [2].

As far as *C. pneumoniae* and renal involvement in childhood are concerned, Tanaka et al in 1999 described a 10-year-old girl with an acute glomerulonephritis associated with pneumonia [3], confirmed by renal biopsy, with a significant increase of her serum title of anti-*Chlamydia pneumoniae* antibodies; Jeffry et al described an acute glomerulonephritis following infection with *Chlamydia psittaci* [4]. Recently, in 2010, Nasser et al. [16] described another 10-year-old child infected by *C. pneumoniae *with acute renal failure. They performed a diagnosis of tubulointerstitial nephropathy after *C. pneumoniae pneumonia*, and they affirmed that *C. pneumoniae* infection may be associated with tubulointerstitial nephropathy and/or rapidly progressive glomerulonephritis whose severity varies in children as in adults. On the consequence, they concluded that early and effective treatment of *C. pneumoniae* infection with macrolide antibiotics usually provides favourable progression of renal function [16].

This presented case shows some interesting aspects not previously reported in the literature; in fact this is, to our knowledge, the youngest patient with renal involvement during course of *Chlamydia pneumoniae* infection and it is the third case of GN-associated-*C. pneumoniae* infection, in childhood described. By the way some of the clinicopathological features and the biopsy specimens presented by our patients are reported in most of the cases of GN associated with *Chlamydia* infections. 

Clinicians should be aware of the potential association between *C. pneumoniae* infection and several extrapulmonar manifestations, and the importance of our report highlights how the early diagnosis and treatment of infection is important in order to avoid renal complications, as *C. pneumoniae* is an eradicable agents.

In conclusion, for this reason in our patient we can hypothesize that the renal involvement is the result of a pathogenic autoimmune mechanism secondary to many factors like immunocomplex depositions or autoantibodies, as shown by the increase, in our patients, of immunologic factors, such as immunoglobulins, C3, C4, CH50, and C1 q inhibitor. Nevertheless further studies are necessary to better understand these pathogenic mechanisms. 

## References

[1] Santos RP, Mayo TW, Siegel JD (2008). Active surveillance cultures and contact precautions for control of multidrug-resistant organisms: ethical considerations. *Clinical Infectious Diseases*.

[2] Iyoda M, Hato T, Matsumoto K (2006). Rapidly progressive glomerulonephritis in a patient with *Chlamydia pneumoniae* infection: a possibility of superantigenic mechanism of its pathogenesis. *Clinical Nephrology*.

[3] Tanaka H, Onodera N, Ito R, Hirano K, Monma N, Waga S (1999). Acute glomerulonephritis associated with pneumonia: a possible *Chlamydia pneumoniae* etiology?. *Pediatrics International*.

[4] Chen M, Schena FP, Wang SP, Grayston JT, Johnson RJ (1998). Role of *Chlamydia pneumoniae* (TWAR) in IgA nephropathy. *Nephron*.

[5] Jeffrey RF, More IAR, Carrington D, Briggs JD, Junor BJR (1992). Acute glomerulonephritis following infection with *Chlamydia psittaci*. *American Journal of Kidney Diseases*.

[6] Davison AM, Davison AM, Cameron JS, Grünfeld JP, Kerr DNS, Ritz E, Winearls CG (1998). Infections-related glomerulonephritis. *Oxford Textbook of Clinical Nephrology*.

[7] Srivastava T, Warady BA, Alon US (2002). Pneumonia-associated acute glomerulonephritis. *Clinical Nephrology*.

[8] Byrom NP, Walls J (1979). Fulminant psittacosis. *The Lancet*.

[9] Wimmer MLJ, Sandmann-Strupp R, Saikku P, Haberl RL (1996). Association of chlamydial infection with cerebrovascular disease. *Stroke*.

[10] Kallenberg CGM (1998). Autoantibodies to myeloperoxidase: clinical and pathophysiological significance. *Journal of Molecular Medicine*.

[11] Iyoda M, Kuroki A, Sugisaki T (2007). *Chlamydia pneumoniae* infection and MPO-ANCA-associated glomerulonephritis. *Nephrology Dialysis Transplantation*.

[12] Campbell LA, Kuo CC (2003). *Chlamydia pneumoniae* and athero- sclerosis. *Seminars in Respiratory Infections*.

[13] Kuo C-C, Grayston JT, Campbell LA, Goo YA, Wissler RW, Benditt EP (1995). *Chlamydia pneumoniae* (TWAR) in coronary arteries of young adults (15–34 years old). *Proceedings of the National Academy of Sciences of the United States of America*.

[14] Marchant AE, Bhandari S, Farr MJ (1995). Reversible acute renal failure associated with *Chlamydia pneumoniae* infection. *Nephrology Dialysis Transplantation*.

[15] Ohsawa I, Ohi H, Endo M (2001). A case of renal involvement in persistent immune activation caused by chlamydial salpingitis. *Virchows Archiv*.

[16] Nasser H, Dib Nehme G, Camoin-Schweitzer M-C, Andre J-L (2010). Acute renal failure following *Chlamydia pneumoniae* pneumonia in a child. *Archives de Pediatrie*.

